# Dying in hospital: socioeconomic inequality trends in England

**DOI:** 10.1177/1355819616686807

**Published:** 2017-01-11

**Authors:** Helen Barratt, Miqdad Asaria, Jessica Sheringham, Patrick Stone, Rosalind Raine, Richard Cookson

**Affiliations:** 1 Senior Clinical Research Associate*,* Department of Applied Health Research, University College London, UK; 2 Research Fellow, Centre for Health Economics, University of York, UK; 3 Senior Research Associate, Department of Applied Health Research, University College London, UK; 4 Professor of Palliative and End of Life Care, Marie Curie Palliative Care Research Department, University College London, UK; 5 Professor of Health Care Evaluation and Head of Department of Applied Health Research, University College London, UK; 6 Professor and NIHR Senior Research Fellow, Centre for Health Economics, University of York, UK

**Keywords:** England, inequalities, palliative care, place of death

## Abstract

**Objective:**

To describe trends in socioeconomic inequality in the proportion of deaths occurring in hospital, during a period of sustained effort by the NHS in England to improve end of life care.

**Methods:**

Whole-population, small area longitudinal study involving 5,260,871 patients of all ages who died in England from 2001/2002 to 2011/2012. Our primary measure of inequality was the slope index of inequality. This represents the estimated gap between the most and least deprived neighbourhood in England, allowing for the gradient in between. Neighbourhoods were geographic Lower Layer Super Output Areas containing about 1500 people each.

**Results:**

The overall proportion of patients dying in hospital decreased from 49.5% to 43.6% during the study period, after initially increasing to 52.0% in 2004/2005. There was substantial ‘pro-rich’ inequality, with an estimated difference of 5.95 percentage points in the proportion of people dying in hospital (confidence interval 5.26 to 6.63), comparing the most and least deprived neighbourhoods in 2011/2012. There was no significant reduction in this gap over time, either in absolute terms or relative to the mean, despite the overall reduction in the proportion of patients dying in hospital.

**Conclusions:**

Efforts to reduce the proportion of patients dying in hospital in England have been successful overall but did not reduce inequality. Greater understanding of the reasons for such inequality is required before policy changes can be determined.

## Introduction

The United Kingdom has been ranked as providing the best ‘quality of death’ and ‘quality of palliative care’ in a recent comparison of 80 countries.^1^ This ranking follows a period of improving end of life care. In England, the national end of life care programme was established in 2004 and a national strategy was published in 2008.^2^ Increasing the ability of individuals to die in the place of their choice is a key aim. Research suggests that up to two-thirds of patients would prefer to die at home, although the proportion varies between studies.^3^ National policy assumes that acute hospital wards are an inappropriate and undesirable place to die.^4^ As a result, the proportion of deaths in hospital has been proposed as a possible indicator of care quality. However, it is recognized that hospital may be the preferred place of care for some patients as their disease progresses.^5^

Place of death depends on a range of factors, including the cause of death, which varies between population groups. Previous analysis using death registration data demonstrated that the proportion of patients dying at home increased in England and Wales from 18.3% in 2004 to 20.8% in 2010.^6^ At the same time, there was a reduction in the proportion of deaths in ‘hospitals and communal establishments for the care of the sick’, including nursing homes.^7^ However, the authors did not consider trends in the care provided to different socio-demographic groups. Another analysis of English mortality data between 2007 and 2009 demonstrated that socioeconomic deprivation is a major determinant of where, when and how people die.^7^ Adjusting for the combined effects of deprivation, age, sex and cause of death, death in hospital was more common in the most deprived areas. In a review of research literature and nationally available data, including the National Survey of Bereaved People in England, 2013, Dixon et al. found that those in more deprived areas were less likely to die at home, despite having similar access to community-based support.^8^ These differences persisted after controlling for age, sex, diagnosis, ethnicity and whether the decedent had a partner. Although place of death has been used as a proxy for care quality in both policy and research,^9^ it is not the most important aspect of ‘a good death’ for patients. In one study, the most important factors were ‘to have my pain/symptoms well controlled’, ‘to not be a burden to my family’ and ‘to have sorted out my personal affairs’.^10^

Equal access for equal need has always been a central tenet of the English NHS.^11^ Although the proportion of deaths occurring in hospital has fallen recently, we do not know whether this figure has improved for all socioeconomic groups and whether the gap between the richest and poorest areas has changed.

The analysis reported here formed part of a larger study, which aimed to develop methods for monitoring NHS equity performance in tackling socioeconomic healthcare inequalities. Using death in hospital as a marker of socioeconomic variation in the quality of end of life care overall, our aim was to assess equity at the small area level. Our objectives were to compare the proportion of patients who died in hospital in the most and least deprived areas and to examine changes in inequalities between 2001/2002 and 2011/2012, defined as the difference between the most and least deprived areas.

## Methods

### Design and setting

This is an ecological study of trends in socioeconomic inequality in the proportion of patients who died in hospital in England between 2001/2002 and 2011/2012. This includes a period of sustained effort by the NHS to reduce the proportion and improve care quality overall. We include the three years before the national end of life programme was launched (2004), and a similar period after the publication of the national strategy (2008). Our study measures socioeconomic inequality between small area populations. The basic geographical unit of analysis was the 2001 ‘Lower Super Output Area’ (LSOA). There are 32,482 of these neighbourhoods in England and Wales, covering approximately 1500 people each (minimum 1000 and maximum 3000).

### Data sources

Our analysis was based on mortality data from the Office for National Statistics (ONS) for financial years 2001/2002–2011/2012. Information about LSOA is contained within the ONS mortality data. We measured the socioeconomic status of each neighbourhood in England using the 2010 Index of Multiple Deprivation (IMD). IMD 2010 overall deprivation rank scores were attributed to each of the LSOAs, which were then ranked according to attributed score. Data are presented according to quintile groups, defined as aggregations of deprivation ranked LSOAs.

### Outcome

Our study indicator measures socioeconomic inequality between small area populations in the proportion of deaths from all causes that occurred in hospital in a given year. The numerator is the number of deaths from any cause, in all ages, in an NHS or other hospital with NHS funding in a given year, using Hospital Episode Statistics (HES). We include deaths from all causes and all ages in both the numerator and the denominator.

### Analysis

Our primary measures of inequality were the slope index of inequality (SII) and relative index of inequality (RII), both based on linear regression analysis at LSOA level.^12^ The proportion of deaths from all causes that occurred in hospital was modelled as a linear function of LSOA level deprivation, entered as a continuous variable scaled from 0 to 1. The SII is the coefficient in this regression; the RII is that coefficient divided by the mean. The SII can be interpreted as the modelled absolute gap between the most and least deprived small area, allowing for the whole socioeconomic gradient; the RII can be interpreted as the proportionate gap relative to the average. We examined inequality in this way because absolute and relative inequality can move in opposite directions when the mean is changing over time.^13^ Linear regression models were computed using pooled data for the first and last year, including interaction terms between year and deprivation, to test for the statistical significance of changes between the beginning and end of the analysis period.

## Results

In England, of 5,260,871 individuals who died between 2001/2002 and 2011/2012, 2,596,945 died in hospital. The proportion of patients dying in hospital decreased from 49.5% to 43.6% and the number of deaths in hospital declined from 247,406 to 199,467 – 47,939 fewer patients each year. However, within this period, between 2001/2002 and 2004/2005, the proportion increased to 52.0%, after which it declined to 2011/2012.

**Figure 1.**
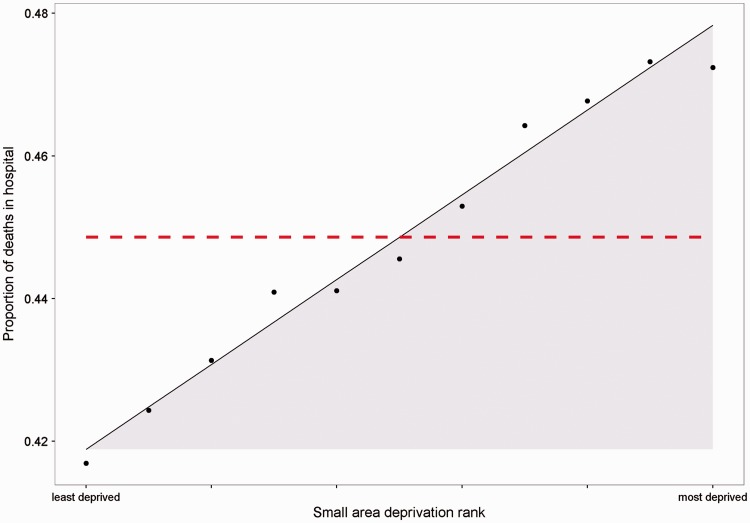
National social gradient in dying in hospital in 2011/2012. Dots represent deprivation decile groups. The slope of the line is the slope index of inequality. The shaded area shows the ‘inequity gap’. The dashed line shows the national average.

There was ‘pro-rich’ inequality throughout the period with a greater proportion of individuals in the most deprived quintiles dying in hospital. In 2011/2012, the estimated inequality gap (SII) was 5.95% of people dying in hospital (95% confidence interval 5.26 to 6.63) between the most and least deprived neighbourhoods (Figure 1). This indicates a relative inequality gap (RII) of 13.6% (95% confidence interval 12.07% to 15.22%) of the national average rate of dying in hospital. Across the social gradient, in that financial year, socioeconomic inequality was associated with 13,593 people (95% confidence interval 12,023 to 15,162) in more deprived areas dying in hospital, rather than other care settings – the area under the curve in Figure 1. In other words, in 2011/2012, there could have been 13,593 fewer deaths in hospital if inequality were reduced to zero.

**Figure 2.**
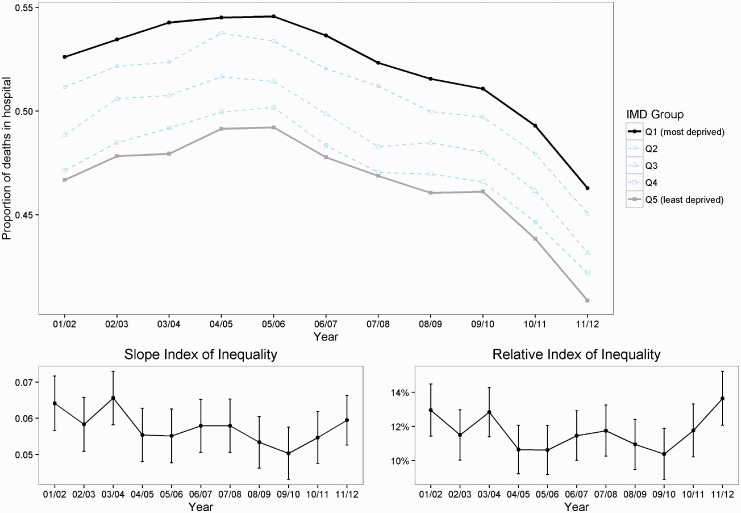
National equity trends in dying in hospital. The solid black line shows the most deprived quintile and the solid grey line shows the least deprived quintile. A positive slope index of inequality indicates a ‘pro-rich’ distribution in absolute terms. A positive relative index of inequality indicates a ‘pro-rich’ distribution in relative terms.

Inequality between deprivation groups persisted throughout the study period. The proportion of patients dying in hospital in the most deprived quintile was at least 5% higher than the least deprived quintile every year (Figure 2). The estimated absolute inequality gap (SII) between the most and least deprived neighbourhoods declined from 6.41% (95% confidence interval 5.66 to 7.16) in 2001/2002 to 5.02% (95% confidence interval 4.30 to 5.76) in 2009/2010, before rising again to 5.95% (95% confidence interval 5.26 to 6.63) in 2011/2012. The relative inequality gap, compared to the national average rate (RII), decreased from 13.0% (95% confidence interval 11.4 to 14.5) in 2001/2002 to 10.4% (95% confidence interval 8.90 to 11.89) in 2009/2010 before rising again to 13.6% (95% confidence interval 12.07 to 15.22) in 2011/2012. As Figure 2 demonstrates, the SII and the RII both fluctuated. However, the confidence intervals overlapped and there was no statistically detectable change in the gap between the most and least deprived neighbourhoods. This is despite the reduction in the average proportion of patients dying in hospital from 2005/2006 onwards.

## Discussion

### Main findings

Using death in hospital as an indicator of the quality of end of life care,^9^ we considered trends in socioeconomic inequality over time. Efforts to improve end of life care in England from 2004 did not reduce inequality, although they did improve quality overall. The proportion of patients dying in hospital rose from 49.5% in 2001/2002 to 52.0% in 2004/2005, after which it decreased to 43.6%. However, substantial ‘pro-rich’ inequality persisted. There was no statistically detectable change in either the absolute gap between the most and least deprived quintiles (SII) or the proportionate gap relative to the national average (RII). End of life care therefore differs from some other areas of care, such as primary care, where reductions in socioeconomic inequality have been achieved.^14^

### Comparison with other studies

To our knowledge, no previous study has examined trends in socioeconomic inequalities in place of death over time. The strength of our study is that it makes use of a comprehensive national data source. HES contain details of all admissions to NHS hospitals in England. In contrast, much of the evidence cited by Dixon et al. in their review was drawn from the National Survey of Bereaved People 2013. Although that is a rich source of data, responses to the questionnaire are socially patterned, as the authors note. Non-response has been shown to be associated with the deceased being male, younger, and area deprivation of place of residence.^8^ Non-response weights are used to minimize the impact of these biases. However, our focus here is on national trends in inequality over time. If disadvantaged people, who are at greater risk of receiving poor quality care, are less likely to participate in a survey, those data are likely to under-estimate socioeconomic inequality. Where selection into a dataset is non-comprehensive and non-random, it is also not possible to tell whether changes over time are real or an artefact of changing patterns of non-response. In our analysis, we considered deaths from all causes. However, areas with different levels of deprivation will also have different diagnostic profiles. For example, cancer is the most common cause of death in the least deprived areas of England, whilst cardiovascular disease and respiratory disease are more common in more deprived areas.^8^ Given the different disease trajectories of these conditions, care pathways will also differ.^6^ For example, a large proportion of deaths from cardiovascular disease occur in hospital rather than in the patient’s home.^15^ As we did not control for diagnosis, there is a risk of potential bias in our analysis. Differences in disease profile between socioeconomic groups should not affect national trends over time, as the epidemiology of common diseases does not change rapidly. They could, in contrast, affect local inequality monitoring, which would require further investigation. Finally, this paper reports an ecological study of trends in socioeconomic inequality. Our unit of analysis was the 2001 ‘Lower Super Output Area’ (LSOA). Although the most deprived LSOAs have the highest rates of death in hospital, we cannot assume that individuals from the most deprived areas are more likely to die in hospital. To fully guard against this ‘ecological fallacy’, individual-level deprivation data would be required. Such data are not available in a form that can be linked to health data in England. Consequently our estimate of the inequality gap is likely to be biased downwards.^16^

There are a number of possible reasons which may explain why socioeconomic inequalities persisted despite reductions in the overall number of hospital deaths. For example, dying out of hospital may not always be achievable or desirable and may not necessarily represent better quality of care for every patient.^8,17^ Access to palliative care is not always straightforward, and dying at home may be less feasible in poor housing.^8^ There are also people for whom home is not their choice location or who change their mind.^3^ Indeed, hospital may be the preferred place of care for many as their disease progresses.^5,9^ We, therefore, cannot conclude that all of the absolute gap between different socioeconomic groups reflects poor quality of care: some of it may reflect good quality care, taking into account the patient’s individual circumstances. The gap may also have persisted because there are substantial inequities in access to palliative care across the UK.^18,19^ There is also known to be pro-rich inequality in the probability of dying in a hospice.^20^

### Implications for research

We have considered socioeconomic inequalities for all conditions. In the future, it would be helpful to examine variations in equity for specific conditions, to identify areas for additional research and potential policy intervention.^17^ For example, there is less evidence about the factors that influence place of death for patients with non-malignant conditions, although their chances of dying at home are generally lower.^3^ Equally, there would be value in investigating potential variations in equity between local NHS areas, and likely explanations for those differences, to inform managers. To understand how to reduce inequalities from a patient’s perspective, we also need to understand how preferences differ between different social groups. Hospitals will almost certainly continue to be a common place of death in this country for the foreseeable future. It is therefore important that actions are taken to improve the quality of the end of life care that they provide.^17^
